# Supplementary material for: Validity of daily self-pulse palpation for atrial fibrillation screening in patients 65 years and older: A cross-sectional study
Source: PLoS Med. 2020 Mar 31;17(3):e1003063. doi: 10.1371/journal.pmed.1003063 (PMC7108684; doi:10.1371/journal.pmed.1003063)
Supplement: S1 Table — STROBE, Strengthening the Reporting of Observational Studies in Epidemiology. (DOCX) [file pmed.1003063.s001.docx]

STROBE Statement—Checklist of items that should be included in reports of ***cross-sectional studies***

|  | Item No | Recommendation | Completion |
| --- | --- | --- | --- |
| **Title and abstract** | 1 | (*a*) Indicate the study’s design with a commonly used term in the title or the abstract | Title and abstract: Cross-sectional screening study for AF among ≥65y patients seeking primary care using pulse palpation vs ECG |
|  |  | (*b*) Provide in the abstract an informative and balanced summary of what was done and what was found | Self-pulse palpation simultaneously with handheld intermittent ECGs three times daily over 2 weeks. |
| Introduction | | | |
| Background/rationale | 2 | Explain the scientific background and rationale for the investigation being reported | Pulse palpation is recommended for single time-point screening for AF but the role of pulse palpation for AF detection has not been validated against simultaneous intermittent ECG |
| Objectives | 3 | State specific objectives, including any prespecified hypotheses | Validity of AF screening by self-pulse palpation simultaneously with handheld ECG recordings 3 times daily for 2 weeks. |
| Methods | | | |
| Study design | 4 | Present key elements of study design early in the paper | Design: Cross-sectional screening study in Swedish primary care. |
| Setting | 5 | Describe the setting, locations, and relevant dates, including periods of recruitment, exposure, follow-up, and data collection | Locations: 4 PCCs around Stockholm, Period: June 2017 to December 2018, Data collection by 1-2 responsible nurses/center. |
| Participants | 6 | (*a*) Give the eligibility criteria, and the sources and methods of selection of participants | Patients who, for whatever reasons, were seeking care at a PCC and who were 65 years of age or older were invited by health personnel to participate. Patients who were interested in participating were then directed to a research nurse in the PCC. Patients with previously known atrial fibrillation or ongoing oral anticoagulation treatment were excluded. |
| Variables | 7 | Clearly define all outcomes, exposures, predictors, potential confounders, and effect modifiers. Give diagnostic criteria, if applicable | The participants completed a questionnaire (Supplement Case Report Form) about stroke risk stratification according to the CHA2DS2-VASc score. Body weight, height, pulse and blood pressure were measured.  The outcome was AF. AF was diagnosed as a 30-second recording with an absolutely irregular rhythm without distinct p-waves. |
| Data sources/ measurement | 8* | For each variable of interest, give sources of data and details of methods of assessment (measurement). Describe comparability of assessment methods if there is more than one group | Screening procedure: The nurse carefully instructed participants in the technique of radial pulse palpation and then checked the participants’ ability to perform self-pulse palpation. The participants were instructed to perform self-pulse palpation at home three times a day over a two-week period immediately followed each time by a 30-second ECG recording using a Zenicor handheld ECG |
| Bias | 9 | Describe any efforts to address potential sources of **bias** | The study nurses recruited relatively younger and probably healthier patients. This could cause selection bias. However, the recruitment of patients with higher morbidity would result in a higher AF detection rate  The validity of pulse palpation vs ECG recording for AF detection was evaluated in the same individual, therefore there no more selection bias. No follow-up and No missing data. Data were collected according to standard. Diagnosis of AF was verified by 2 cardiologist. On unclear ECG recordings, individuals was further investigated by 5 days continuous ECG monitoring. |
| Study size | 10 | Explain how the study size was arrived at | Screening periods varied from 9 to 15 months in the four participating centers. The screening ended at all centers in December 2018 as the prespecified sample size had been included. |
| Quantitative variables | 11 | Explain how quantitative variables were handled in the analyses. If applicable, describe which groupings were chosen and why | Please see the paragraph “Statistical analyses” in the Methods of this manuscript. |
| Statistical methods | 12 | (*a*) Describe all statistical methods, including those used to control for confounding | Multivariate logistic regression analyses were conducted to analyze the independent predictors for AF detection. |
|  |  | (*b*) Describe any methods used to examine subgroups and interactions | Such methods have not been used in this study. |
|  |  | (*c*) Explain how missing data were addressed | No missing data. |
|  |  | (*d*) If applicable, describe analytical methods taking account of sampling strategy | Not applicable |
|  |  | (*e*) Describe any sensitivity analyses | Such analyses have not been used in this study. |
| Results | | | |
| Participants | 13* | (a) Report numbers of individuals at each stage of study—eg numbers potentially eligible, examined for eligibility, confirmed eligible, included in the study, completing follow-up, and analysed | Please see Table 1.  Of 8412 PCC-visitors, 626 patients had previously known AF and the remaining 7786 individuals were eligible but minority of them were invited for AF screening and 1010 individuals were screened. |
|  |  | (b) Give reasons for non-participation at each stage | Of visitors without AF, 4–28% were screened, where only some of these visitors were invited to be screened, depending on the capacity of health staff to screen. No real estimate of agreement to screening was made but the recruiting health personal considered the agreement rate to be around 80–90%. |
|  |  | (c) Consider use of a flow diagram | Please see table 2 instead flow diagram |
| Descriptive data | 14* | (a) Give characteristics of study participants (eg demographic, clinical, social) and information on exposures and potential confounders | The median age of the screened individuals was 72.1 years and 61.6% were female. |
|  |  | (b) Indicate number of participants with missing data for each variable of interest | No missing data |
| Outcome data | 15* | Report numbers of outcome events or summary measures | 27 (2.7%, CI 1.8–3.9%) new cases of AF were detected. Anticoagulant treatment was initiated by the patient’s general practitioner in 26 (96% CI 81–100%) of new AF cases. Please see “AF detection” paragraphs in the results of this manuscript. |
| Main results | 16 | (*a*) Give unadjusted estimates and, if applicable, confounder-adjusted estimates and their precision (eg, 95% confidence interval). Make clear which confounders were adjusted for and why they were included | For the newly detected AF cases, the median age was 76.4, male domination (70.4%) and more prevalent heart failure. Age and male gender were independent predictors for detection of new AF cases with an odds ratio (95% CI) of 1.14(1.07–1.21) and 4.46 (1.9–10.43), respectively. Please see table 3 and fig 1 |
|  |  | (*b*) Report category boundaries when continuous variables were categorized | No continuous variable was categorized. |
|  |  | (*c*) If relevant, consider translating estimates of relative risk into absolute risk for a meaningful time period | This was not done. |
| Other analyses | 17 | Report other analyses done—eg analyses of subgroups and interactions, and sensitivity analyses | Please see paragraph “Validity of pulse palpation” in the results of this manuscript and tables 4 and 5. |
| Discussion | | | |
| Key results | 18 | Summarise key results with reference to study objectives | 1-Low sensitivity (56%) and high specificity (81%) of individual’s self-pulse palpation three times daily for two weeks compared to simultaneous intermittent ECG recording for AF detection.  2-Five times more AF detection (27 new cases) through intermittent ECG over two weeks compared to only five new AF cases detected at baseline ECG as single-time-point measurement.  3-Age and male gender were independent predictors for AF detection.  4-Anticoagulants could be initiated in almost all (26) new cases of AF in which non-vitamin K antagonist oral anticoagulants were initiated in 25 cases. |
| Limitations | 19 | Discuss limitations of the study, taking into account sources of potential bias or imprecision. Discuss both direction and magnitude of any potential bias | 1-PCCs were not randomly selected for recruitment. This could affect the reproducibility of our results.  2-A minority of patients who visited PCCs were invited to the screening and there was no real estimate of participation among the invited patients. The study nurses recruited relatively younger and probably healthier patients. This could cause selection bias.  3-Ectopic heart beats could be felt as an irregular pulse and this could reduce the specificity of pulse palpation for AF.  No other potential bias suspected in this study. |
| Interpretation | 20 | Give a cautious overall interpretation of results considering objectives, limitations, multiplicity of analyses, results from similar studies, and other relevant evidence | This is the first study evaluating intermittent self-pulse palpation simultaneously with intermittent ECG for AF screening.  Key results 2-4 confirm the results of previous studies. |
| Generalisability | 21 | Discuss the generalisability (external validity) of the study results | As Limition-1, generalisability could be affected by non-random selection of screening centers. |
| Other information | | | |
| Funding | 22 | Give the source of funding and the role of the funders for the present study and, if applicable, for the original study on which the present article is based | This work was supported by the Swedish Heart-Lung Foundation, Boehringer Ingelheim, Bayer CropScience and the Pfizer Foundation. The funders had no role in study design, data collection and analysis, decision to publish, or preparation of the manuscript |

*Give information separately for exposed and unexposed groups.

**Note:** An Explanation and Elaboration article discusses each checklist item and gives methodological background and published examples of transparent reporting. The STROBE checklist is best used in conjunction with this article (freely available on the Web sites of PLoS Medicine at http://www.plosmedicine.org/, Annals of Internal Medicine at http://www.annals.org/, and Epidemiology at http://www.epidem.com/). Information on the STROBE Initiative is available at www.strobe-statement.org.
