# Supplementary material for: Validity of daily self-pulse palpation for atrial fibrillation screening in patients 65 years and older: A cross-sectional study
Source: PLoS Med. 2020 Mar 31;17(3):e1003063. doi: 10.1371/journal.pmed.1003063 (PMC7108684; doi:10.1371/journal.pmed.1003063)
Supplement: S1 Text — (DOCX) [file pmed.1003063.s003.docx]

## Case Report File

Personal data

Name _____________________________

Telephone number ____________________________

Primary care center ____________________________

Inclusion date ____________________________

Examination

Pulse palpation Regular Irregular

Blood pressure _________ /_________ mm Hg

Height _________ cm

Body weight _________ kg

Do you have?

Diabetes mellitus Yes No

Heart failure Yes No

Previous history of stroke/TIA Yes No

Hypertension Yes No

Previous myocardial infarction or peripheral artery dis. Yes No

Gender Female Male
