# Supplementary material for: Validity of daily self-pulse palpation for atrial fibrillation screening in patients 65 years and older: A cross-sectional study
Source: PLoS Med. 2020 Mar 31;17(3):e1003063. doi: 10.1371/journal.pmed.1003063 (PMC7108684; doi:10.1371/journal.pmed.1003063)
Supplement: Attachment — Submitted filename: Response to Reviewers.docx [file pmed.1003063.s004.docx]

We thank the reviewers for the careful reading of our manuscript. All comments raised by the reviewers have been carefully addressed. We believe that the review process has greatly improved our manuscript and that the comments were excellent and helpful. We hope that the present version is acceptable. The manuscript has completely been reviewed again by a language review agency. We have discussed the comments and we revised our manuscript in order to be suitable for publication as we explain below:

Comments from the reviewers:

Reviewer #1: I confine my remarks to statistical aspects of this paper. The basic approach is fine, but I have some issues to resolve before I can recommend publication.

Line 39 - Should "patients" here be "recordings"?

**Authors’ response: Thanks for remark. This was wrong written and it has been corrected as requested**.

Line 150 The usual power is 0.80. Why did the authors choose 0.75? (It isn't necessarily wrong to do so, but it needs justification).

**Authors’ response: This study is mainly an explorative study. We aimed mainly to study the validity of self-pulse palpation. Detection of significant difference in AF detection was a secondary aim and we had limited study resources. Therefore, it was not very important to choose a high power. However, the calculated sample size was 955 and we decided to screen at least 1000 individuals. Finally, our screening results showed statistically significant difference between AF detected by intermittent ECG and AF detected by first single ECG.**

Lines 153-155 How were the CIs calculated (there are several methods). **Authors’ response:** **It has been clarified as** “**Calculations with exact Clopper-Pearson confidence intervals were used for sensitivity and specificity while the standard logit confidence intervals were used for the predictive values.”**

Line 182 Give the interquartile range.

**Authors’ response: Thank you for your advice. It has been given.**

Fig 1 - pie graphs are not good. Here, a simple table would be fine. Also, why were two of the N's approximate?

**Authors’ response: It has been replaced by a table according to your suggestion.**

Fig 3 - this is hard to read. If exact age is available, then it would be better to use two overlaid density plots - one for men and one for women - with lines for screened and general population for each. If age is only available in categories, then two mosaic plots could be presented, or these could be combined into one three-way mosaic plot.

**Authors’ response: It has been replaced as requested.**

Fig. 4 Stacked bar charts are not good and 3-D makes them worse. I'm not sure what to suggest here. Maybe just a table. Maybe a mosaic plot. Maybe a bar plot in 2D with the bars adjacent to each other.

**Authors’ response: It has been replaced by table.**

Reviewer #2: Ghazal et al have report a cross-sectional screening study in patients >65 years of age visiting primary care centers and taking part in AF screening. Screening was performed with intermittent ECG recordings three times per day for a period of 2 weeks and simultaneous pulse palpation. A total of 1010 patients participated in the study and 27 (2.7%) new cases of AF were detected in 311 ECG recordings, of which the pulse was palpated as irregular in 77 patients (25%). 187 individuals without AF felt an irregular pulse on least one occasion. The specificity per measurement occasion and per individual was (98%). They conclude that AF screening using self-pulse palpation three times daily for two weeks has lower sensitivity compared with simultaneous intermittent ECG. Thus, it may be better to screen for AF using intermittent ECG without stepwise screening using pulse palpation.

The study idea is important, since silent AF is a common problem and stroke is too often its 1st manifestation (PLOS One 2016;11:e0168010). The execution of the study is adequate and the sample size is sufficient for the present study questions. The report is generally well-written.

**Authors’ response: Thank you for your valuable comments and to the reference that you suggested about silent AF. We have now referred to this study.**

The weakness of this study is the unclear selection process of study patients weakening the generalizability of the results.

**Authors’ response: We agree with you about the unclear selection of participants. We cannot exactly describe who included versus those were not. When we compare our screening population with Swedish general population, our screening is somewhat younger. This can indicate that our study patients were healthier. Our patients were recruited from several primary care centers with different study nurses which can cause individuals selections bias. Finally, administrative issues could have been played an important role.**

The sensitivity of pulse palpation was lower than expected showing that this method is not suitable for every elderly subject. In an earlier study, elderly (> 75 years of age) subjects the accuracy of pulse palpation after a training given by study nurse was: sinus rhythm 97%, extra beats 74.3%, slow AF 81.8% and fast AF 91.9% when assessed with the help of anatomic human arm model programmed with various rhythms ( Scand J Prim Health Care 2017;35:93-98). The main problem with pulse palpation is the primary motivation and ability (Parkinson, severe arthrosis in fingers etc) and later the motivation to continue the habit of regular palpation.

**Authors’ response: Thank you for referring to this study.** **We have now referred to this study.**

Did the study nurses evaluate the ability of subjects to measure their pulse after the training session?

**Authors’ response: Yes, it was done in every patients.**

What were the predictors for missing AF with pulse palpation?

**Authors’ response: We believe that missing to check the pulse and low variability of irregular pulse could have contributed to miss AF.**

Did the subjects really palpate the pulse and did they report heart rate during each recording?

**Authors’ response: We did not studied the heart rate. There is risk that the subject missed to check their pulse. However, when we founded AF on ECG with remark as regular pulse, we contacted the subject by phone to recall pulse-palpation state and to remind.**

What was the reason for misdiagnosis of AF?

**Authors’ response: Probably the occurrence of ectopic heart beats.**

Reviewer #3: This nicely executed study looks at an important issue, which to my knowledge has not previously been assessed. It aims to determine if self-performed pulse palpation is as accurate at detecting unknown AF as a hand-held ECG, over a period of 2-weeks. This is an important issue to clarify, as the economics of hand-held devices for AF detection are much higher than just educating people to take their own pulse. Overall, this is a nicely presented paper. I believe the methodology is appropriate and the study is powered sufficiently to answer the research question.

There are a few minor things in the paper that I think need to be clarified, and a few places where the English grammar may need a little editing.

**Authors’ response: Thank you for your valuable comments. The manuscript has now been subjected to a professional English language editing.**

Line 46-47 - I am not quite sure that the last sentence of the abstract conclusion means - it reads as if you are suggesting NOT to do step-wise screening, but I do not think that is the case.

**Authors’ response: Yes, we do not suggest stepwise screening using self-pulse palpation as it has low sensitivity missing many cases of AF. Thus, it is inferior to intermittent ECG. However, if there is limited access to intermittent ECG, then stepwise screening may be an alternative.**

2. Line 60 - "who searched care" - should this be "who sought care"

**Authors’ response: It has been changed to “who were seeking care”**

3. Line 78 - The initial use of the acronyms AF and AC are not defined on their first use in the body of the manuscript. Additionally, it is more common for OAC to be used rather than AC.

**Authors’ response: It has been changed as requested. One of our AF patients received LMWH, therefore we used AC instead of OAC.**

4. Line 102 states you recruited in Stockholm County and then line 104 states you screened OUTSIDE Stockholm County.

**Authors’ response: Thank you for this remark. It was wrong written and it has been corrected by deleting “in Stockholm”**

5. Lines 118- 130 - More information is required in the methods section under the screening protocol to describe: how the participants recorded the pulse palpation results; how the ECG and pulse palpation results were assessed to determine AF or no AF; and what other tests were done; how symptoms were determined and recorded; what follow up occurred if AF was identified and how the patient was advised they had AF; who determined the treatment; and how OAC was prescribed.

**Authors’ response: Thank you for these advices. This paragraph in the screening procedure has been rephrased.**

6. Line 147 - there is an updated meta-analysis of screening published by the same authors which may be better to quote instead of reference 4. DOI: 10.1371/journal.pmed.1002903

**Authors’ response: This new meta-analysis is a good study but we calculated our sample size in the planning stage based on previous meta-analysis then.**

7. Line 166-167 - Can you explain this a little better - I am not quite sure what this sentence means and it may be a source of bias in the selection of practices that could be worth noting in the limitations.

**Authors’ response: We agree with you. This sentence was unclear therefore we omitted it.**

I think a further point for the discussion is that the pulse palpation performed by patients themselves is significantly less sensitive and specific than pulse palpation performed by health professionals. There are some previously older studies that have also identified this**.**

**Authors’ response: We found only one study about the sensitivity of self-pulse palpation for AF detection showing higher sensitivity by self-pulse palpation than pulse palpation performed by health professionals when a group of elderly was trained using an anatomical model to check pulse irregularity. This model was thus experimental and cannot be compared to the clinical situation.**
