# Supplementary material for: Validity of daily self-pulse palpation for atrial fibrillation screening in patients 65 years and older: A cross-sectional study
Source: PLoS Med. 2020 Mar 31;17(3):e1003063. doi: 10.1371/journal.pmed.1003063 (PMC7108684; doi:10.1371/journal.pmed.1003063)
Supplement: Attachment — Submitted filename: Response to Reviewers.docx [file pmed.1003063.s005.docx]

Comments from the reviewers:

Reviewer #1: The authors have addressed my concerns and I now recommend publication.

Reviewer #2: I have no further comments. The authors have responded adequately to my previous comments.

Reviewer #3: The authors have done a good job in revising the manuscript, but I note that many comments were not addressed within the manuscript itself - they were just addressed in the response to reviewers. I would suggest that the authors go back through the original reviewer comments and check that each point raised by the reviewers has been answered within the manuscript, unless there is a good reason for why it should not be altered, in which case the response should indicate that nothing was altered because xxx reason.

**Authors’ response to Reviewer-3: Thank you for your valuable comments. We are really sorry that we did not address some comments within the manuscript previously. Our response for the previous and new comments as follow:**

*For example, Reviewer 1 requested that an explanation be provided for the choice of 0.75 statistical power rather than 0.8. This point should be explained in the manuscript, not just to the reviewer.

**Authors’ response: Reviewer-1 was satisfied with our previous response. However, we have now clarified it in lines 187-188 as “We chose 0.75 power because of limited resources.”**

The methods still require some further clarification - mostly with information that has been brought up in the original reviewer comments - eg:

* how did the nurses identify the eligible patients - were they patients that they were seeing for a clini8cal consultation or did the doctors refer their patients to them

**Authors’ response:** **We have rephrased the text in lines 126-128 as follow “Patients who, for whatever reasons, were seeking care at a PCC and who were 65 years of age or older were invited by health personnel to participate. Patients who were interested in participating were then directed to a research nurse in the PCC.”**

* were the practices offered any incentives for participation, or given anything for the training - did the nurses get accreditation points etc

**Authors’ response:** **We have added these points in lines 129 and 134-135 as follow “No incentives were given for participation.” And “No incentives or accreditation points were given for this training”**

* did the nurse test/check the patients ability to pulse palpate after training

**Authors’ response:** **Yes. Pulse-palpation ability was tested after training. We have rephrased the text in lines 141-143 as follow “The nurse carefully instructed participants in the technique of radial pulse palpation and then checked the participants’ ability to perform self-pulse palpation.”**

* when were people asked to do the recordings at home - and how often

**Authors’ response:** **As it stated in the manuscript line 144“at home three times a day”. We now further clarified it in lines 146-148 as “We did not specify which time of day the participants should perform self-pulse palpation. However, we preferred them to do it at different times of the day.”**

* Were they asked to do the pulse and ECG at the same time

**Authors’ response: We have clarified it in line 145 as** “**immediately followed each time by a 30-second ECG recording”**

* How did they record their pulse palpation result (did they have a diary to write it in?)

**Authors’ response:** **As it stated in the manuscript “The Zenicor device has a button that participants can use to note when their pulse felt irregular”. We further clarified it in line 154 as “during each ECG recording”**

* How was the pulse palpation and ECG recordings compared

**Authors’ response:** **It has been clarified in lines 189-190 as follow “we compared the results of each pulse palpation with the results of a simultaneous ECG recording.”**

* If they had symptoms with the pulse palpation how did they record these

**Authors’ response:** **The participants asked about their symptom only when the ECG showed AF.** **It stated in the manuscript in line 157**

* Who reviewed the ECGs and was this done remotely or with the patient

**Authors’ response:** **It has been clarified in lines 154-156 as “The responsible author is an experienced family doctor who interpreted the ECG register remotely each day”**

* Did the patient come back into the centre at the end of the period of time for a final review and to return the device

**Authors’ response:** **It has been clarified in lines 162-164 as “Participants with no detected AF returned the handheld ECG devices at the end of the screening with no scheduled follow up and were then notified by post about their ECG results.”**

* The methods should indicate that the family doctor was responsible for prescription of the AC - this is mentioned later in the manuscript, but not in the methods

**Authors’ response:** **It has been clarified in lines 160-161 as “Thus, the patient’s family doctor was responsible for the initiation of AC.”**

Limitations

* I don't understand what is meant by the first limitation. It is vague and needs rewording.

**Authors’ response:** **It has been changed to “PCCs were not randomly selected for recruitment” in line 348-349**

* The authors mention in their comments that the different study nurses at each site can introduce a selection bias, but this is not mentioned in the limitations section

**Authors’ response:** **We added this point in the limitation lines 353-356 as “The study nurses recruited relatively younger and probably healthier patients. This could cause selection bias. However, the recruitment of patients with higher morbidity would result in a higher AF detection rate.”**

*The authors state in their response that this was not step-wise screening, but the conclusion of the abstract refers to stepwise screening - this is confusing. Why is step-wise screening introduced as a concept here?

**Authors’ response:** **Stepwise screening defined as pulse palpation followed by ECG recording when the pulse is irregular. We would not recommend it since the pulse palpation has low sensitivity. We have now clarified this point in lines 321-323**

*I would suggest that the authors go back through the original reviewer comments and check that each point raised by the reviewers has been answered within the manuscript, unless there is a good reason for why it should not be altered, in which case the response should indicate that nothing was altered because xxx reason.

**Authors’ response:** **As requested from Reviewer-3, we rechecked the previous comment from other reviewers. We previously explained our response to a comment from Reviewer-2 without including the response in the manuscript.**

“What were the predictors for missing AF with pulse palpation?” **We believe that missing to check the pulse and low variability of irregular pulse could have contributed to miss AF.** **Reviewer-2 was satisfied with our previous response.** **Nothing was altered in the manuscript because we did not evaluate these issues in our study.**

**We previously explained our response to a comment from Reviewer-2 without including the response in the manuscript. “**Did the subjects really palpate the pulse and did they report heart rat0e during each recording?” **We did not studied the heart rate. There is risk that the subject missed to check their pulse. However, when we founded AF on ECG with remark as regular pulse, we contacted the subject by phone to recall pulse-palpation state and to remind. Reviewer-2 was satisfied with our previous response. However, we have now further clarified this point in line 156 by adding the text** **“reminding to check their pulse”**

**Reviewer-3 previously preferred to refer to some previously older studies about the sensitivity of self-pulse palpation.** “I think a further point for the discussion is that the pulse palpation performed by patients themselves is significantly less sensitive and specific than pulse palpation performed by health professionals. There are some previously older studies that have also identified this.” **This is interesting but unfortunately, we could not found these studies.**

**Nothing was altered in the manuscript because we could not found these studies.**

**As previous comment from Reviewer-3:** Line 182 - there is an updated meta-analysis of screening published by the same authors (doi: 10.1160/TH13-02-0165) which may be better to quote instead of reference doi: 10.1160/TH13-02-0165. Epub 2013 Apr 18. **Thus, Reviewer-3 preferred to change our reference of a meta-analysis used in sample size calculation in the statistical analyses to an updated one published September 25, 2019. We calculated sample size in the planning phase year 2016 therefore we used the meta-analysis published 2013. We started our screening 2017 and ended December 2018.**

**Nothing was altered in the manuscript because the new study was published after our planning phase. However, both studies reported comparable AF detection rate of 1.4% vs 1.44% in old and updated one respectively.**
